# iCOSSY: An Online Tool for Context-Specific Subnetwork Discovery from Gene Expression Data

**DOI:** 10.1371/journal.pone.0131656

**Published:** 2015-07-06

**Authors:** Ashis Saha, Minji Jeon, Aik Choon Tan, Jaewoo Kang

**Affiliations:** 1 Department of Computer Science and Engineering, Korea University, Seoul, Korea; 2 Department of Medicine/Medical Oncology, University of Colorado Anschutz Medical Campus, Aurora, Colorado, United States of America; 3 Interdisciplinary Graduate Program in Bioinformatics, Korea University, Seoul, Korea; University of Padova, ITALY

## Abstract

Pathway analyses help reveal underlying molecular mechanisms of complex biological phenotypes. Biologists tend to perform multiple pathway analyses on the same dataset, as there is no single answer. It is often inefficient for them to implement and/or install all the algorithms by themselves. Online tools can help the community in this regard. Here we present an online gene expression analytical tool called iCOSSY which implements a novel pathway-based COntext-specific Subnetwork discoverY (COSSY) algorithm. iCOSSY also includes a few modifications of COSSY to increase its reliability and interpretability. Users can upload their gene expression datasets, and discover important subnetworks of closely interacting molecules to differentiate between two phenotypes (context). They can also interactively visualize the resulting subnetworks. iCOSSY is a web server that finds subnetworks that are differentially expressed in two phenotypes. Users can visualize the subnetworks to understand the biology of the difference.

## Introduction

Genes act in networks to exert various biological functions. Altered gene expressions in specific subnetworks could lead to different biological states, including progression of diseases such as cancer. These gene expressions could be monitored using high-throughput genome-wide microarrays or next-generation transcriptome sequencing (RNA-seq). However, conventional differential expression analyses that focus on single gene markers are less effective in identifying such subnetworks within a network. Recent results have demonstrated that pathway-based analyses are more effective than single gene analyses in identifying these subnetworks for disease classifications [1, 2]. Researchers are now focusing on developing computational methods for pathway-based analyses in an attempt to uncover these complex interactions within disease subnetworks [3–7]. Gene interactions help researchers generate new hypotheses about complex traits development.

Khatri et al. classified pathway-based analyses into three broad categories: 1) over-representation analysis approaches that typically count differentially expressed genes in a pathway, 2) functional class scoring approaches that leverage coordinated expressions in functionally related genes, 3) pathway topology-based approaches that utilize pathway topology [8]. Gene Set Enrichment Analysis (GSEA) [6], one of the most popular pathway-based analysis tools, represents one of the tools in the second category. Our recently proposed algorithm COntext-Specific Subnetwork discoverY (COSSY) [9] represents one of the tools in the third category. COSSY is a non-greedy algorithm that can be used to discover closely interacting subnetworks that can discriminate two phenotypes using gene expression profiles. COSSY first splits a network depending on its topology, and then ranks all the subnetworks according to their coordinated differential gene expressions.

Signaling Pathway Impact Analysis (SPIA) [10] represents one of the methods in the third category and uses gene expression profiles and pathway topology to find significant pathways. SPIA takes into account the proportion of differentially expressed genes in a pathway and the genes’ positions in the pathway (e.g. gene-to-gene interactions in the pathway). SPIA does not consider the size of the pathway but rather the links in the pathway. The pathways are then ranked according to their scores and returned as results. Pathway-Express [11] also returns a ranked list of static pathways and provides links to the KEGG website. On the other hand, iCOSSY finds subnetworks that are made up of differentially expressed genes in a pathway. Therefore, subnetworks identified by iCOSSY are inherently much smaller than pathways identified by SPIA. Furthermore, iCOSSY provides seamless visualization for top subnetworks without using any third-party tools. iCOSSY can interactively merge (or stitch) multiple top subnetworks; this can help generate new hypotheses for further experimental testing. The original COSSY, SPIA and Pathway-Express do not provide this additional function.

Graphite web [12] is an online web server that analyzes pathways and visualizes networks using gene expression profiles (both microarray and RNA-seq). It implements five different gene set analysis methods (including SPIA and GSEA) for three model organisms using KEGG and Reactome pathway databases. Graphite web employs graphite [13] to convert pathway topology to a gene network. Differentially expressed genes identified by one of the gene set analysis methods are visualized as highlighted nodes in the gene network. However, no subnetwork extractions were performed by Graphite web. Similar to Graphite web, iCOSSY also constructs a gene network from a pathway database. However, instead of performing gene analysis based on individual pathways in gene networks, iCOSSY first divides a network into multiple subnetworks and performs analysis in those subnetworks. It ranks the subnetworks based on the biological context of interest as shown by the gene expression changes within the members of the subnetworks. The final results are a ranked list of subnetworks, where users can visualize or interactively merge the subnetworks. Graphite web does not provide the merging (or stitching) function.

Pathway analyses are more complex than conventional single gene analyses. Users require a significant amount of knowledge and expertise to perform such analyses. Moreover, biologists use many algorithms to analyze their datasets. Installing software packages or applications requires significant efforts. Online tools can be helpful for the community in this regard. Here, we present an online tool called iCOSSY that improves the original COSSY algorithm. iCOSSY is more robust and can better interpret subnetworks. Using iCOSSY, users can readily perform context-specific subnetwork discovery analysis on their own expression data without needing any programming skills. They can also visualize the resulting subnetworks on a web interface. Users can explore and merge different subnetworks in iCOSSY, which can help them generate new hypotheses from their data.

## Materials and Methods

### Context-Specific Subnetwork Discovery (COSSY) Algorithm

In this work, we have developed an online, user-friendly tool that discovers from gene expression data coordinated differentially expressed genes and their associations in molecular interaction networks. It is an improved version of our recently proposed COSSY algorithm [9]. In brief, COSSY first splits an interaction network into smaller, tightly connected subnetworks which we refer to as Molecular Interaction Subnetworks (MISs). Then, it ranks MISs according to the expression patterns of their corresponding molecules. Each MIS gets an entropy score that signifies its rank. The lower the score, the higher the rank, i.e., the more important the MIS. To calculate the entropy score of an MIS, COSSY uses the five most differentially expressed genes as the representative genes of the MIS. The differential expression of a gene is measured using a modified version of Welch’s t-statistic score based on the interquartile range (IQR). If multiple MISs overlap significantly, they are merged together to form a new MIS. Once the representative genes of the MIS are selected, COSSY clusters all the samples using the representative genes’ expression values. The entropy score is then defined based on the proportion of samples of different classes in each cluster. See [9] for details.

### Improvements in iCOSSY

We made COSSY more robust and easier to use. First, inspired by [14], we use fuzzy rank normalization (by default) instead of quantile normalization followed by a z-transformation. In this fuzzy rank normalization, gene expressions of a sample are normalized to a number between 0 and 1. The top *θ*_1_% (here 5%) and bottom *θ*_2_% (here 15%) of genes of a sample are normalized to 1 and 0, respectively. The middle gene expressions are normalized proportionally. Here, normalized expressions 0 and 1 represent low and high expressions, respectively. If a gene is highly expressed (or lowly expressed) in different samples, biologists tend to consider the gene is expressed (or unexpressed) even though its absolute expression scales differ. The fuzzy rank normalization step is consistent with this fact. Moreover, the step removes outliers from the dataset, as previously described [14].

Second, we use the p-value of the original Welch’s t-test to select the representative probeset of an MIS (by default). As there are no outliers in the dataset (which have been removed in the normalization step), we do not need to use the IQR-based modification in iCOSSY. Furthermore, whereas the original COSSY implementation merges the overlapped MISs to protect the original network information, we do not.

Third, most importantly, we apply 10-fold cross validation to identify the most consistently highly ranked MISs from the gene expression data. The input data are randomly divided into 10 sets. In each fold, one set is held out and the top MISs are listed out applying the regular subnetwork discovery algorithm to the remaining nine sets. Ideally, a consistent MIS should appear in the top MIS list in all 10 folds. Here, we count the frequency of each MIS (in how many folds an MIS appears in the top MIS list), and choose the most frequent MISs to build the final model. If multiple MISs have the same frequency, we order them according to their entropies. This process reduces the chances of incorrectly ranking networks and allows iCOSSY to identify the most robust MISs from the gene expression data. To validate this point, we tested the robustness of iCOSSY and the original COSSY. iCOSSY turned out to be substantially more robust than the original COSSY. The test result is given in the supplementary file (S1 Supporting Information).

**Fig 1:**
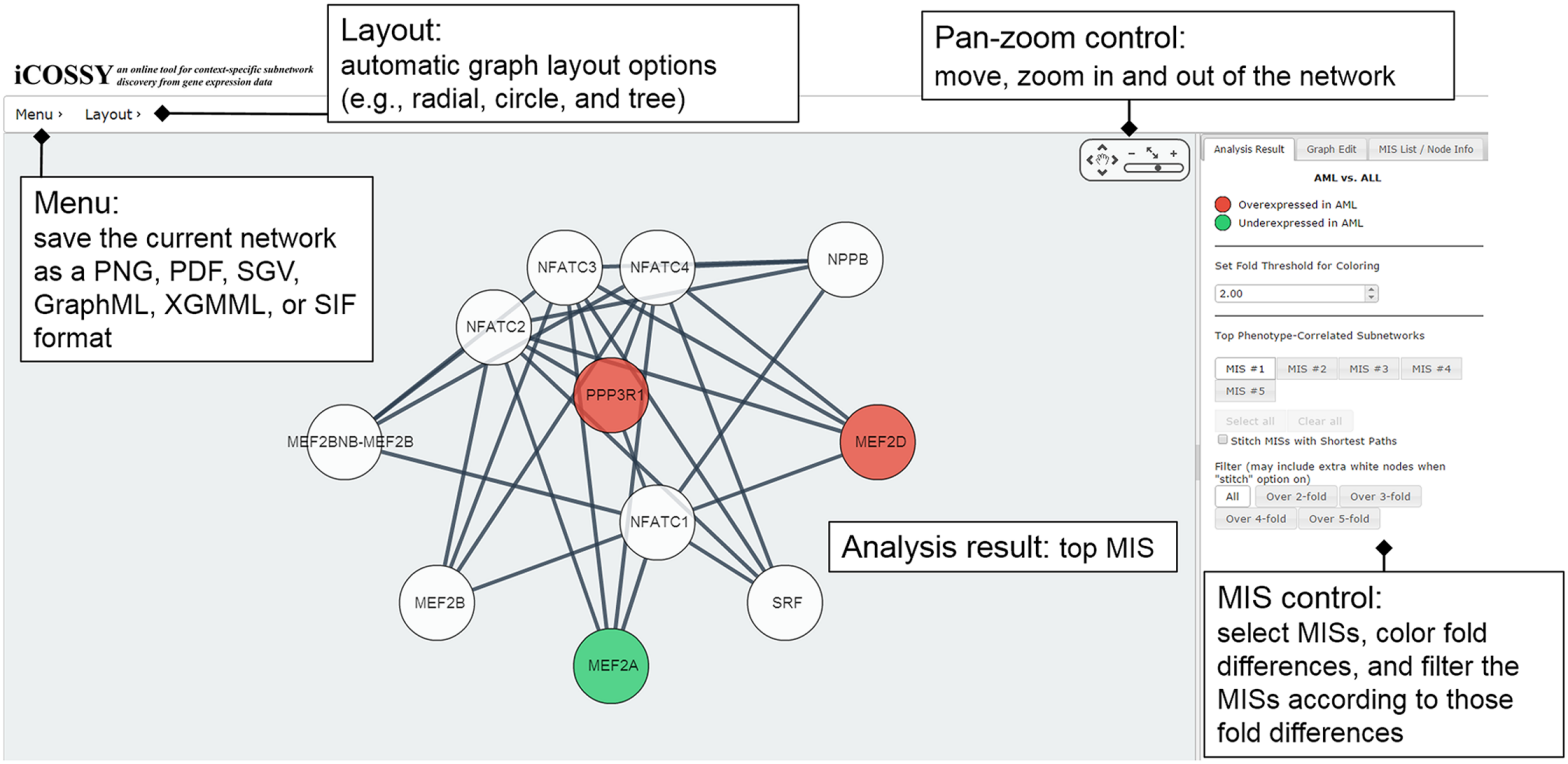
Subnetwork visualization. A top MIS in the leukemia dataset. Red and green colored nodes represent over- and under-expressed genes, respectively.

Last, we implemented a new visualization interface for iCOSSY, and developed an automatic approach that “stitches” different MISs together to form a new hypothesis for down-stream analysis and validation. iCOSSY visualizes the analysis results as a network where nodes and edges represent genes and interactions, respectively. It allows users to intuitively interpret the results. Differentially expressed genes are represented as red (high) or green (low) nodes. As a default, iCOSSY shows the top MIS result in the web interface (Fig 1). As illustrated in Fig 1, there are checkboxes, on the right side of iCOSSY’s web interface, for customizing the results. Using them, users can view different top MIS results. The lower checkbox allows users to stitch multiple MISs together. To stitch subnetworks together, iCOSSY uses the molecular interaction network that the user chose in the beginning (e.g., KEGG, STRING or Pathway API). If two isolated subnetworks can be linked by extra edges, iCOSSY adds them and provides one large network. Otherwise, iCOSSY searches the shortest paths between each pair of genes from each subnetwork based on the molecular interaction network. The shortest paths are ordered by the lengths of the paths, fold differences, and the PageRank scores of the nodes in the paths. Then, we add a few top paths and link the subnetworks. The length of the shortest path is 4 at most.

### Software Used

The core COSSY algorithm is implemented in R. We used several R packages including biomaRt [15], igraph [16], jsonlite [17], Rserve [18], plyr [19], KEGG.db [20], and KEGGgraph [21]. We also used PHP, Apache2 web server, MySQL, and Cytoscape [22] for network visualization.

### System Requirements

The iCOSSY web interface runs on modern internet browsers with JavaScript and Flash enabled. The website is best viewed in the latest versions of Chrome (31+), Safari (5.1+), and Internet Explorer (11+, with the compatibility option turned off). We have tested iCOSSY in the following operating systems: Windows, Linux, and Mac OSX.

## Results and Discussion

### iCOSSY: an online tool

**Fig 2:**
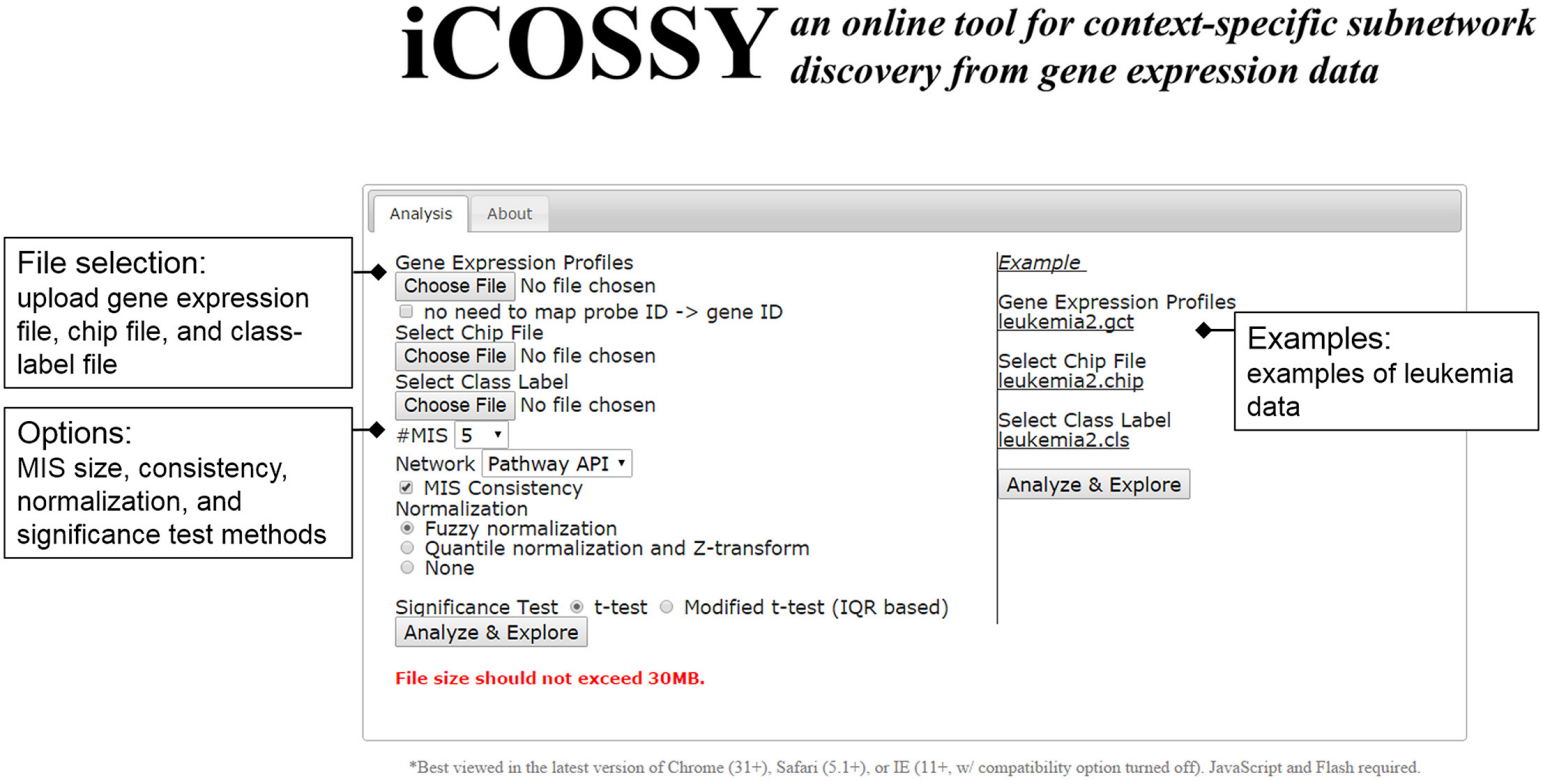
Web interface. The most common scenario involves a user uploading a gene-expression file, a class-label file, and if necessary a chip file, and then clicking the “Analyze & Explore” button to get the results. The website provides the user a link that shows the results once the analysis is completed.

We have newly developed a web server called iCOSSY, available at http://icossy.korea.ac.kr. Here, users can upload their own gene expression profile datasets along with known labels, and can identify the important subnetworks differentially expressed between the labels. The web interface is shown in Fig 2. Network visualization is important for generating new hypotheses. Users can interactively visualize subnetworks returned by iCOSSY analysis (see Fig 1). They can view networks as separate entities or as a whole. iCOSSY automatically stitches multiple subnetworks using KEGG, STRING, or Pathway API. The nodes (genes) are colored according to their expression patterns. Users can also freely edit graphs and save networks for later use.

### Inputs

#### Gene Expression Profiles and Class Labels

Gene expression profiles and class labels, both of which are the most important tab delimited files, contain gene expression data collected from a number of samples and the phenotype labels of the samples, respectively. iCOSSY supports popular .gct and .cls file formats, as described in [23]. In a gene expression file, each row represents a probeset (microarray) or a gene (RNA-seq) across all the samples, and each column represents a sample across all the probesets or genes. Phenotype labels of the samples are written in a class file.

#### Profile Type and Chip File

The web service can handle multiple types of profiles. If profiles contain microarray data, probes are mapped to genes using a chip file (see [23] for format). If profiles contain RNA-seq data, the “description” column of the.gct file must contain the gene map. We should note that HGNC gene symbols are used for mapping.

#### Molecular Interaction Networks

The iCOSSY website currently supports three types of network databases: 1) Kyoto Encyclopedia of Genes and Genomes (KEGG)—Release: 71.0 [24]; 2) Search Tool for the Retrieval of Interacting Genes/Proteins (STRING)—Version 9.1 [25]; and 3) Pathway API—an aggregated database that combines databases from WikiPathways, Igenunity, and KEGG [26].

#### Advanced Configurations

Although iCOSSY selects a set of default configurations, advanced users can customize their analyses to generate useful hypotheses. They can set the configuration attributes, the number of MISs, the significance test, the MIS consistency, and the data normalization methods.

### A Workflow Example

**Fig 3:**
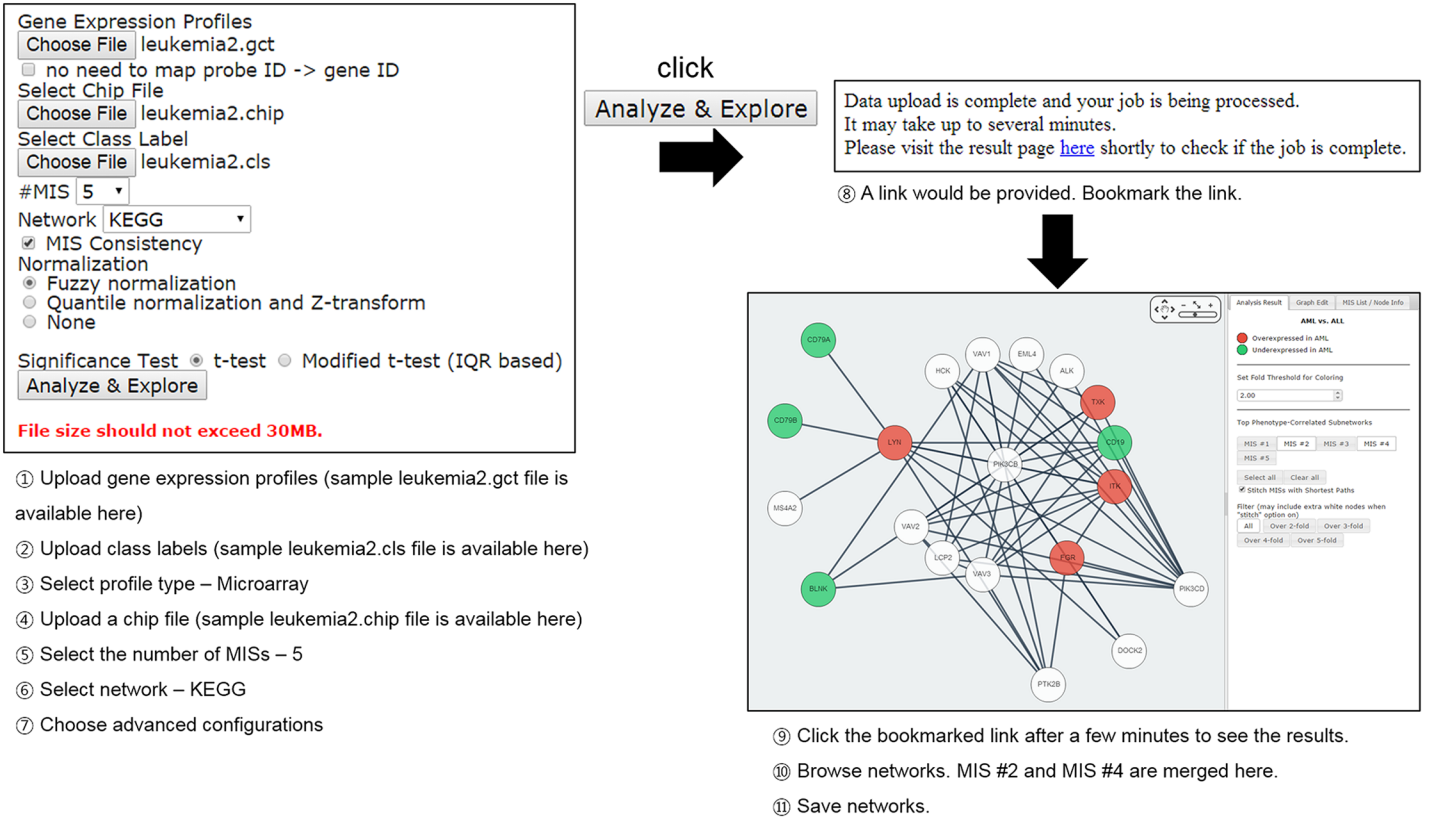
A workflow example that uses a leukemia dataset.

Fig 3 explains a workflow example using a leukemia dataset (AML vs. ALL) [27]. iCOSSY highlights subnetworks that are most correlated with the phenotypes. Users can explore and combine the subnetworks and construct their own networks (hypotheses) using the stitch and filter options. Finally, users can save their networks as images (e.g., PNG, SVG, and PDF) or as standard graph markup formats (e.g., XGMML, GraphML, and SIF).

### Advantages

The newly developed iCOSSY web tool outperforms the R package published earlier in [9] for the following reasons: 1) The R package could analyze only a handful of predefined datasets. iCOSSY allows users to upload and analyze their own datasets. 2) As iCOSSY has been implemented as a web service, users can readily use it without worrying about software installation. 3) iCOSSY allows users to view the important subnetworks in an interactive way. This would help users observe a subnetwork’s expression pattern of a specific phenotype and generate new hypotheses about the underlying molecular mechanisms. 4) iCOSSY is an improved version of COSSY. Users can now find consistently differentially expressed networks, decreasing the chances of getting a random network and increasing the confidence of the analysis. Users can also preprocess data as they desire.

## Conclusions

We presented iCOSSY, an online tool for context-specific subnetwork discovery from gene expression data. Using the iCOSSY web tool, users can readily perform COSSY analysis on their own data and visualize important subnetworks. We believe that this will be a useful and informative tool for users, especially biomedical researchers, who analyze their data to generate new hypotheses.

## Supporting Information

S1 Supporting Information Robustness test results for iCOSSY and COSSY. (PDF)
