# Supplementary material for: iCOSSY: An Online Tool for Context-Specific Subnetwork Discovery from Gene Expression Data
Source: PLoS One. 2015 Jul 6;10(7):e0131656. doi: 10.1371/journal.pone.0131656 (PMC4492968; doi:10.1371/journal.pone.0131656)
Supplement: S1 Supporting Information — (PDF) [file pone.0131656.s001.pdf]

Supplementary Data. iCOSSY: an online tool for context-specific subnetwork discovery from gene expression data

Robustness test results

To validate that iCOSSY is more robust to (i.e., less sensitive to) noise in input data than the original COSSY, we performed a robustness test. For example, to measure the robustness of iCOSSY on the leukemia dataset (of 72 samples), we first generate 72 different leave-one-out sub-datasets each of which consists of 71 samples (left one sample out). We then compute the top-five MISs using the sub-datasets and compare them with the top-five MISs computed using the full dataset. We finally average the overlaps.

The results are shown in the table below. As shown in the results, iCOSSY is much more robust to variations in the input than the original COSSY.

		iCOSSY			COSSY		
dataset	size	max	min	avg	max	min	avg
leukemia	72	5	4	4.65	4	0	1.81
leukemia2	48	5	3	4.69	3	0	1.35
dlbcl	77	5	2	4.25	5	1	3.10
cns	34	5	2	3.38	3	0	1.29
lung	181	5	3	4.20	5	2	3.78
prostate1	102	5	2	3.75	4	1	2.85
prostate3	33	5	2	4.18	5	0	1.97
gcm	280	5	3	4.37	5	0	2.38
